# Progress in the Expression, Purification, and Characterization of Recombinant Collagen

**DOI:** 10.3390/bioengineering13020159

**Published:** 2026-01-28

**Authors:** Youlin Deng, Jiyao Kang, Xiaoqun Duan, Yingjun Kong, Weiquan Xie, Dongjie Lei, Tingchun Wang, Guifeng Zhang

**Affiliations:** 1 School of Pharmacy, Guilin Medical University, Guilin 541199, China; dp18777896160@163.com (Y.D.); robortduan@163.com (X.D.);; 2 State Key Laboratory of Biopharmaceutical Preparation and Delivery, Institute of Process Engineering, Chinese Academy of Sciences, Beijing 100190, China; 3 Guangzhou Boji Medical & Biotechnological Co., Ltd., Guangzhou 511300, China

**Keywords:** recombinant collagen, expression system, purification techniques, characterization

## Abstract

Synthesized by expressing natural collagen sequences in specific hosts, recombinant collagen exhibits multiple advantages, encompassing a higher content of bioactive domains, enhanced antioxidant activity, the absence of viral pathogens, favorable hydrophilicity, reproducible production, and low immunogenicity. Consequently, it has found extensive use in applications ranging from biomaterials and pharmaceuticals to skincare. This review systematically explores various expression systems for recombinant collagen, including those utilizing *Escherichia coli*, *Pichia pastoris*, plants, insect baculovirus, and mammalian cells. It provides a detailed comparison of their differences and commonalities in terms of production efficiency, post-translational modification capability, and cost-effectiveness. Key separation and purification techniques for recombinant collage-notably precipitation, affinity chromatography, ion-exchange chromatography, and gel filtration chromatography are further introduced, with an in-depth analysis of the applicable scenarios and purification outcomes for each method. Finally, the review comprehensively summarizes the characterization methods for both the physicochemical properties and biological functions of recombinant collagen. For physicochemical properties, techniques covered include scanning electron microscopy, micro-differential thermal analysis, circular dichroism spectroscopy, SDS-PAGE, mass spectrometry, and Fourier-transform infrared spectroscopy. For biological functions, the focus is on its roles and the corresponding assessment methods in processes such as cell proliferation, migration, adhesion, and wound healing. Building upon this comprehensive overview, current challenges facing recombinant collagen are identified, and future directions are proposed, emphasizing the need to reduce R&D costs, refine testing methods for cosmetic products, and improve safety evaluation protocols to advance the field.

## 1. Introduction

Collagen is the most abundant protein in the extracellular matrix and connective tissues of vertebrates, widely distributed in connective tissues, skin, bones, visceral cells, the cornea, and other areas. It accounts for approximately 25–30% of the total protein in the human body. As a structural protein, collagen plays a crucial role in providing a scaffold for cells, influencing cellular activities such as adhesion, migration, proliferation, and differentiation [1]. It is also closely associated with physiological processes such as joint lubrication, wound healing, calcification, and blood coagulation. Traditional methods of collagen extraction typically involve acid hydrolysis, alkaline hydrolysis, or enzymatic digestion to isolate collagen from animal sources such as skin (e.g., pig, fish, donkey, cattle, or sheep skin), bones, and placenta [2].

**Figure 1:**
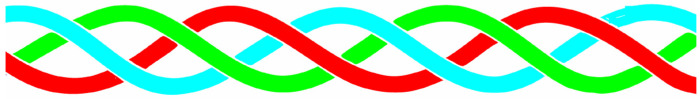
The characteristic triple-helix structure of collagen.

**Figure 2:**
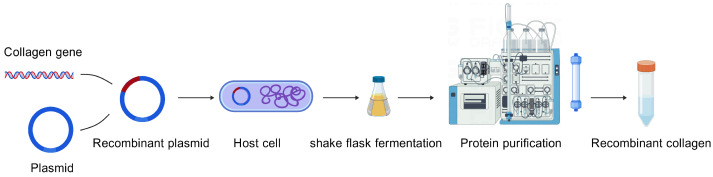
The formation of recombinant collagen. Created with BioGDP.com [6].

**Figure 3:**
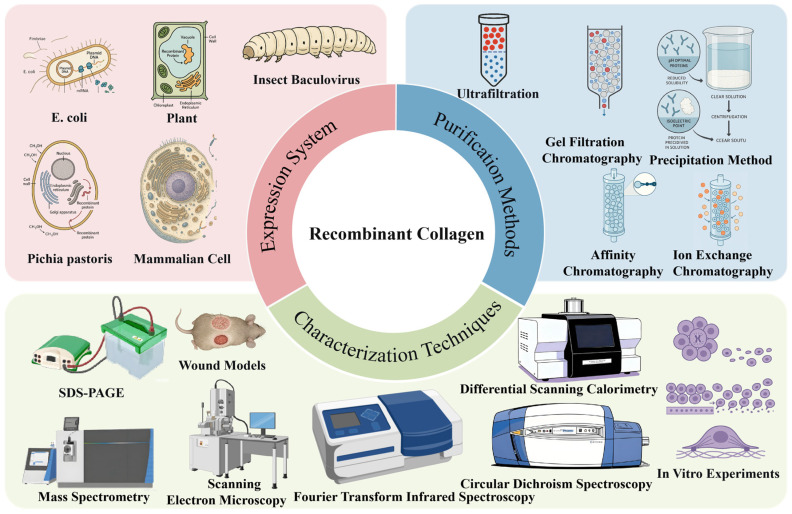
Expression System, Purification, and Characterization of Recombinant Collagen.

Recombinant collagen refers to a protein produced by constructing recombinant expression vectors based on human or model organism collagen gene sequences through gene synthesis technology [3,4]. These vectors are introduced into suitable host organisms (such as *E. coli*, yeast, or animal and plant cells) for expression. The resulting product, obtained through purification and modification, retains the sequence, structure, and function of natural collagen. The triple helix structure of collagen consists of three polypeptide chains (Figure 1). The core of recombinant collagen lies in the Gly-X-Y repeating amino acid sequence derived from natural collagen. Researchers can precisely design and express functional domains containing this repeating sequence, ensuring the recombinant product folds correctly and forms a stable triple-helical structure. The production of recombinant collagen involves expression of the encoded sequence in selected host cells, followed by purification of the expressed product (Figure 2). Compared to traditionally extracted collagen, recombinant collagen offers several advantages, including higher bioactivity regions, superior antioxidant capacity, freedom from viral contamination risks, good water solubility, stable production processes, and low immunogenicity [5]. In recent years, the production of recombinant collagen has transitioned from scientific research to industrial applications, and it is now widely used in fields such as biomedicine and cosmetic skincare. This review focuses on the expression systems, purification methods, and characterization techniques for recombinant collagen (Figure 3).

## 2. Recombinant Expression Systems

The development of recombinant collagen dates back to the 1980s, spanning a history of over 40 years. In 1980, Booth et al. [7] first demonstrated the feasibility of in vitro recombinant expression of human type I and type III procollagen using skin cells. In 1995, the Institute of Biomedical Science at Japan’s Terumo Corporation pioneered the laboratory-scale expression of recombinant human type III collagen using an insect cell-baculovirus system, marking the first international achievement of its kind [8]. By 2001, Professor Fan’s team [9] at China’s Northwest University made a breakthrough in prokaryotic expression by successfully expressing recombinant human type I collagen using an *Escherichia coli* system. To date, common prokaryotic expression systems include *E. coli*, Bacillus subtilis, and Lactococcus lactis, with *E. coli* being the most mainstream system. Eukaryotic expression systems encompass yeasts (including *Pichia pastoris*, *Saccharomyces cerevisiae*, *Hansenula polymorpha*, *Kluyveromyces marxianus*, *Yarrowia lipolytica*, etc.), plants (including tobacco and corn), insects (including silkworms and fruit flies), and mammalian cells (including Chinese Hamster Ovary (CHO) cells, HEK293 cells, and mouse mammary cells) (Table 1).

Due to the short cycle time and low cost of *E. coli*, coupled with the eukaryotic modification capabilities and high-density fermentation advantages of *P. pastoris*, these two systems have become the most widely used expression systems for industrial production. Many current *E. coli* systems can achieve yields exceeding 1 g/L [11,38,39]. In earlier studies, the low expression levels were primarily due to the focus on the complex mechanism of co-expressing functional full-length human collagen with hydroxylase. This resulted in intricate product structures with demanding post-translational modification requirements, combined with the use of unoptimized gene sequences and rudimentary fermentation techniques [13]. Strategies such as co-expressing translation elongation factors with protease-deficient engineering or implementing multi-level optimization including gene copy number increase and chaperone co-expression have enabled high-yield production of recombinant collagen in *Pichia pastoris* [26,36]. While the endogenous hydroxylation capacity of *P. pastoris* is limited, introducing heterologous hydroxylases offers an engineering solution. However, this modification may come at the cost of reduced overall protein expression. Mammalian cell systems show a broad range of expression levels, from an early peak of 8 g/L to recent yields typically around 300 mg/L. Both plant-based and insect expression systems generally exhibit relatively low expression levels, and no significant progress in this regard has been reported in recent years. The choice of expression system for recombinant collagen critically influences its yield, post-translational modifications, and production costs (Table 2).

### 2.1. E. coli Expression System

*E. coli* holds low cost and high efficiency as its core competencies in recombinant collagen expression, making it the preferred host for the large-scale production of low to medium molecular weight collagen [40]. Particularly in scenarios where production yield is prioritized over ultimate structural activity, it offers irreplaceable cost-performance advantages compared to yeast and mammalian cells, and is also the most widely used host bacterium within prokaryotic systems.

However, unlike eukaryotic hosts, *E. coli* lacks the corresponding enzymes. It requires either co-expression of exogenous hydroxylases or post-expression modification to enhance the hydroxylation levels of prolyl and lysyl residues, thereby making the structural characteristics of the expressed recombinant collagen similar to those of natural human collagen. The percentage of proline hydroxylation is closely related to the formation of the collagen triple helix. Zhu et al. [41], by comparing various prolyl hydroxylases for co-expression of recombinant human type III collagen, found that L593 yielded the highest percentage of proline hydroxylation. Cell adhesion was significantly enhanced for the recombinant human collagen (co-expressed with BaP4H and L593). Furthermore, when expressing structurally complex exogenous proteins, *E. coli* often leads to protein misfolding and aggregation into insoluble inclusion bodies. Han et al. [42] dissolved the expressed insoluble recombinant collagen in guanidine hydrochloride and then refolded it into its native conformation under controlled redox conditions. The selection of a suitable expression vector along with the addition of affinity tags can significantly increase protein expression yield and promote soluble expression. Xie et al. [11] utilized pBV220 as the expression vector, which increased both the expression level and solubility of the recombinant collagen, achieving a yield as high as 1.88 g/L. The fusion tag TrxA was used to benefit the soluble expression of COL17 variants [43].

In summary, utilizing prokaryotic systems like *E. coli* to express recombinant collagen with full biological activity still requires overcoming a series of key technological bottlenecks, ranging from efficient expression to correct modification and assembly. The primary challenge lies in addressing the innate lack of post-translational modifications. *E. coli* itself lacks the mammalian P4H) system and cannot catalyze the formation of hydroxyproline, which is essential for a stable triple-helical structure. Although strategies involving the co-expression of key modifying enzymes offer a potential solution, this pathway still faces significant obstacles in practical application: the exogenous P4H enzyme complex often exhibits low expression levels and poor assembly efficiency of its subunits within the prokaryotic system. At the level of protein folding and quality control, a key challenge is ensuring that the hydroxylated collagen chains fold efficiently and correctly within the intracellular environment to form thermodynamically stable homo/heterotrimeric high-order structures. Collagen molecules that fail to fold correctly or in a timely manner are easily recognized and degraded by the host protease system, thus severely impacting the final yield of the functional protein.

### 2.2. Pichia Pastoris Expression System

As the most widely used heterologous protein expression host among yeasts, *Pichia pastoris* offers advantages such as rapid growth, no risk of endotoxins, capability for post-translational modifications, secretory expression capacity, and ease of genetic manipulation [44]. Consequently, it has become one of the most frequently employed host systems in both research and industrial production of recombinant collagen. Commonly used *P. pastoris* strains for heterologous protein expression in scientific and industrial contexts include GS115, MC100-3, Y-11430, X-33, KM71, and SMD1168 [45,46]. Among these, GS115 is the most widely utilized in recombinant collagen expression due to its high genetic stability and superior secretion efficiency.

Using *P. pastoris* GS115 as the host, Chen et al. [47] developed a fusion protein expression system for recombinant human-like collagen (HLC) and human bone morphogenetic protein. They achieved successful expression and scaled-up production, resulting in a recombinant collagen that combines the structural characteristics of HLC with the stability of human bone morphogenetic protein. In another study, Fang et al. [48] co-expressed the recombinant human type III collagen α1 chain and P4H in *P. pastoris* GS115, successfully producing hydroxylated human recombinant collagen.

Despite its significant potential for industrial applications, *P. pastoris* still faces challenges in practical use. Keizer et al. [49] observed that excessively large recombinant procollagen molecules may accumulate in the endoplasmic reticulum post-translationally rather than being secreted extracellularly. Additionally, the relatively long expression cycle of *P. pastoris* imposes higher stability requirements on the secreted target proteins.

From the perspective of host system selection logic, *P. pastoris* has gained greater preference over *E. coli* among recombinant collagen researchers in recent years. The primary reason lies in its ability to circumvent key limitations of *E. coli.* Proteins expressed in *E. coli* require additional endotoxin removal steps, which increase purification costs and process complexity. In contrast, *P. pastoris* poses no endotoxin risk, making it better aligned with the core safety requirements for medical-grade recombinant collagen.

### 2.3. Plant Expression System

Plant expression systems primarily utilize species such as tobacco, barley, and corn, enabling large-scale and low-cost production of recombinant collagen. However, their production yields remain relatively low [50].

In early studies, Ruggiero et al. [15] employed tobacco plants as an expression system to produce human homotrimeric type I collagen. They successfully expressed disulfide-bonded trimers that folded into stable homotrimeric triple-helical recombinant collagen, though the yield was only 30 mg/kg. Later, Merle et al. [50], using a tobacco expression system and co-expressing with the hydroxylase P4H, achieved the production of recombinant hydroxylated homotrimeric collagen, with yields ranging from 50 to 100 mg/kg. Stein et al. [51] further utilized tobacco to express recombinant type I collagen under the action of two enzymes—human P4H and lysyl hydroxylase 3 (LH3). The resulting collagen formed a thermostable triple-helical structure and exhibited biological functions similar to those of human collagen, with yields reaching 200 mg/kg. Although yields have improved from the initial 30 mg/kg to 200 mg/kg over years of development, and the thermal stability of the triple-helical structure has been enhanced, the production level still lags significantly behind those achieved in *E. coli* and *P. pastoris* systems.

In another approach, Xu et al. [52] co-expressed the recombinant human type I collagen α1 chain with the α and β subunits of recombinant human prolyl 4-hydroxylase (rP4H) in transgenic corn seeds, successfully obtaining recombinant collagen with high hydroxyproline (Hyp) content. However, due to the use of the globulin-1 gene promoter to drive endosperm-specific expression of the recombinant human type I collagen α1 chain, the average yield was only 4 mg/kg. In contrast, seeds without rP4H co-expression achieved an average yield of 12 mg/kg of full-length recombinant human type I collagen α1 per kilogram of seeds. This indicates that although co-expression with hydroxylase enhances the hydroxylation content of recombinant collagen, it leads to a reduction in yield.

In the absence of co-expression with exogenous hydroxylases, transgenic plants offer little advantage in terms of collagen hydroxylation. Additionally, plant growth cycles are multiple times longer than bacterial fermentation, and this production method carries the risk of transgene contamination [51]. As a result, research in this area has remained largely stagnant in recent years. In previous studies, large-scale plant-based production of recombinant collagen has mostly been limited to intracellular expression in whole-plant cultivation systems. To date, there have been no reports on achieving high-yield production of target proteins using plant in vitro culture techniques. Moreover, the expression cycle lasts from several weeks to several months [52].

### 2.4. Insect Baculovirus Expression System

The baculovirus-insect cell expression system leverages the comprehensive post-translational modification machinery of insect hosts to enable precise folding and processing of complex proteins, resulting in expression products with three-dimensional conformations highly similar to their natural counterparts.

Lamberg et al. [31] found that under the action of recombinant P4H, the Pro-alpha 1 chain of human type III collagen expressed in Sf9 cells contained substantial amounts of 4-hydroxyproline and formed triple-helical molecules, while the expression level of type III procollagen also increased. Tomita et al. [8] efficiently expressed the type III collagen precursor pro-alpha1(III) chain in insect cells using a recombinant baculovirus system. A small portion of the expressed pro-alpha1(III) chains formed normal disulfide-bonded procollagen III and were secreted into the culture medium, while the majority formed trimeric molecules with similar thermal stability but were neither disulfide-bonded nor secreted, and the hydroxylation of prolyl residues was relatively low. The same issue was observed by the team two years later in their study on recombinant type I collagen, where they expressed full-length human type II collagen cDNA in silkworm larvae [53]. SDS-PAGE and Western blot analyses indicated that the collagen α chains formed a triple-helical structure, and activity assays confirmed that the recombinant protein exhibited bioactive properties [23].

Through enzymatic modification or direct utilization of live insects, this system can effectively synthesize collagen with a triple-helical structure and bioactivity, providing a feasible pathway for the production of functional recombinant collagen. However, challenges such as inconsistent hydroxylation levels, extended expression cycles, and a high proportion of non-ideal trimers remain prevalent, limiting its industrial application.

### 2.5. Mammalian Expression System

Compared to traditional bacterial or yeast expression systems, mammalian cells offer greater complexity and can produce collagen with structures and activities more closely resembling those of human collagen [40]. They exhibit superior hydroxylation capacity and are capable of generating triple-helical collagen.

Wagner et al. [54] utilized HEK293 and HT1080 cells to secrete full-length triple-helical hrColX molecules. However, the secreted collagen showed relatively low hydroxylation levels, with a hydroxyproline-to-proline ratio of only 0.25. After co-transfection with the α and β subunits of human P4H, this ratio increased to nearly 0.5, leading to enhanced thermal stability of the hrColX triple-helical structure. Fukuda et al. [55] employed CHO cells to co-express two α1(IV) and one α2(IV) chains, which assembled into a stable triple-helical structure resistant to pepsin digestion. Under neutral conditions, the RhCol1 fibrils demonstrated excellent self-assembly capability, forming stronger collagen fibers. Wang et al. [20] expressed the full-length human type I collagen α1 chain in CHO cells, achieving a hydroxyproline content of 9.1% and a yield of 300 mg/L. The following year, the same team used this expression system to produce full-length human type II collagen, reaching a yield of 188.73 mg/L [24].

The core advantage of mammalian cell expression systems over prokaryotic, yeast, and insect cell systems lies in their ability to provide a post-translational modification environment most similar to human cells. This enables efficient production of recombinant products with structures and functions highly analogous to native human proteins, offering superior human compatibility. However, compared to microbial expression systems, mammalian cell platforms have significant drawbacks, primarily their considerably longer expression cycles. This stems from three interrelated factors: First, animal cells inherently grow slowly, requiring substantially longer cultivation times than bacteria or yeast, extending the entire production process from inoculation to harvest to several weeks. Second, animal cells are highly sensitive to culture conditions—such as temperature, pH, dissolved oxygen, nutrients, and shear stress—necessitating reliance on sophisticated and expensive bioreactors with complex control strategies. Finally, the high cost of culture media further limits their application, as these media are not only compositionally complex but also require the addition of serum or costly serum-free supplements. Overall, mammalian cell systems offer irreplaceable advantages in producing clinically compatible recombinant collagen, despite their high cost and long cycle.

## 3. Purification of Recombinant Collagen

Among various purification strategies, techniques such as precipitation [25], affinity chromatography [56], ion exchange chromatography [57], and gel filtration chromatography [58] constitute critical pathways from crude extracts to highly pure recombinant collagen, leveraging their unique separation mechanisms and technical advantages (Table 3). As a classical purification method, precipitation enables rapid concentration and impurity removal through precipitation effects. Although its resolution is limited, it establishes a foundation for subsequent fine purification steps. Affinity chromatography, relying on specific biomolecular interactions, achieves ultra-high throughput enrichment in a single step, making it particularly suitable for refining recombinant tag-fused proteins. Ion exchange chromatography exploits differences in protein surface charges to accomplish intermediate purification with high loading capacity and ease of scalability. Gel filtration chromatography, based on the principle of molecular size exclusion, facilitates buffer exchange and separation of multimers while preserving biological activity. Since no single purification method can adequately meet the high purity requirements of the final product, practical applications often necessitate the sequential integration of multiple purification techniques.

### 3.1. Precipitation Method

**Figure 4:**
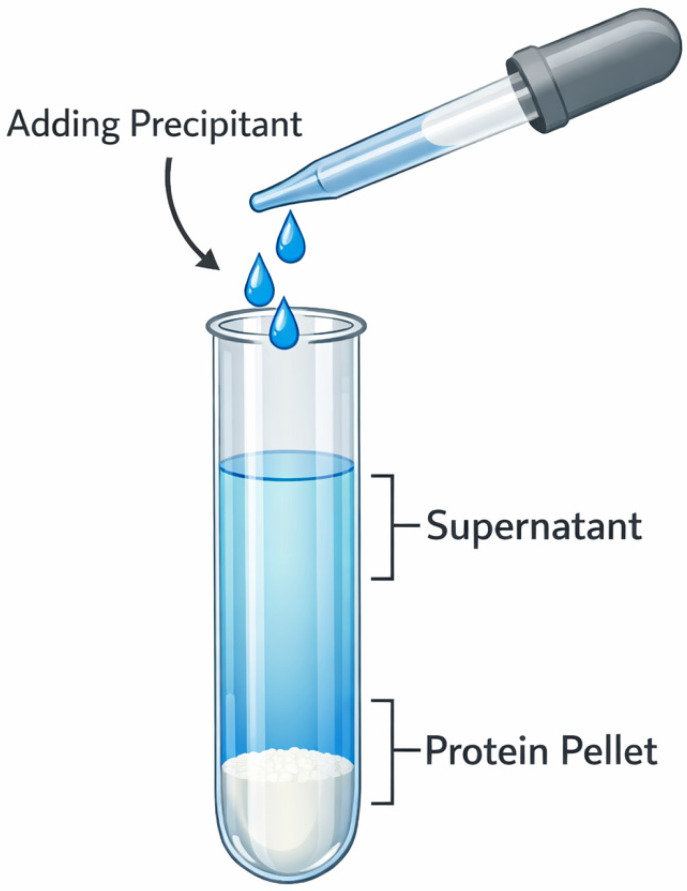
Schematic diagram of the Precipitation Method.

Commonly used precipitation methods for protein purification include salting-out [67], acid precipitation [68], and isoelectric point precipitation [69]. Among these, salting-out precipitation is often employed as an initial crude purification step to achieve preliminary separation and enrichment of the target protein, typically using neutral salts such as (NH_4_)_2_SO_4_, NaCl, and Na_2_SO_4_. The addition of high concentrations of salt ions disrupts the hydration layer of protein molecules, reducing their solubility and causing precipitation (Figure 4).

Salt precipitation of an acidic corn seed extract, without pepsin pretreatment, yielded collagen devoid of foldon with 100% recovery and >70% purity, with pepsin pretreatment, the yield was 94.0% with a purity of 76.5% [67]. Initial purification by NaCl salting-out and pH precipitation yielded rhColIV-E at only 45.37% purity. Subsequently, ion-exchange chromatography combined with ultrafiltration desalination significantly enhanced the purity to 94.32% [70]. In contrast, Yan et al. [25] successfully achieved 92% purity of rhCLA through a crude separation method combining alkaline and acidic precipitation. Subsequent purification via cation exchange chromatography increased the final purity to over 98%. These results indicate that precipitation can serve as an effective primary purification method, but its separation efficiency is limited. Combining precipitation with chromatographic techniques is crucial for achieving high-purity collagen preparation.

Precipitation offers advantages such as low cost, simple operation, and high scalability. However, its purification efficacy heavily depends on the intrinsic properties of the target protein, the solvent environment, and the composition of impurity proteins. Consequently, its resolution and selectivity are limited, making it difficult to obtain high-purity products in a single step. To meet application requirements, precipitation often needs to be integrated with chromatographic techniques, forming a complementary purification strategy. Current limitations of this method include: relatively harsh operating conditions that may cause protein denaturation or activity loss; insufficient separation capability for complex samples; and generally poor recovery rates and reproducibility. Additionally, subsequent steps such as desalting and redissolution are often required, which increases process complexity and time costs.

### 3.2. Affinity Chromatography

**Figure 5:**
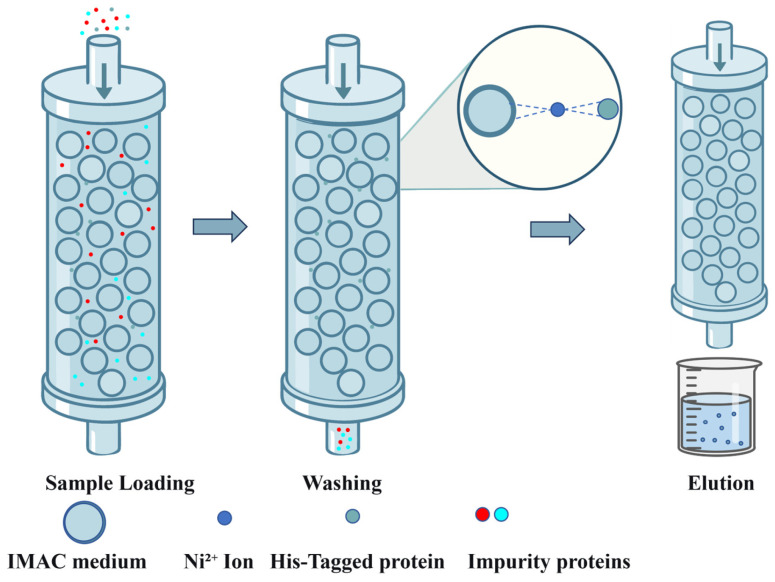
Schematic Diagram of His-Tag Affinity Chromatography.

Affinity chromatography is one of the key techniques for purifying recombinant collagen. Its core advantage lies in the specific interaction between the target protein and the ligand, enabling highly efficient capture of the target protein (Figure 5). The use of gentle elution conditions helps preserve the structural integrity and bioactivity of the recombinant collagen, making this method particularly suitable for applications requiring high purity. Commonly used tags include His (histidine) and SUMO (Small Ubiquitin-like Modifier).

Gahlawat et al. [71] incubated the supernatant with a Ni-NTA affinity chromatography column at 4 °C for one hour, removed nonspecifically bound proteins using a low-concentration imidazole buffer, and finally eluted the target protein with a high-concentration imidazole gradient, obtaining recombinant protein of certain purity. Teng et al. [72] reported that the purity of hCOL1A1 protein purified using Ni-NTA affinity chromatography was only about 50%. Further purification, including enzymatic digestion and two-step ion exchange chromatography (both anion and cation), was required to achieve a purity exceeding 95%. The recombinant type III collagen was purified using Ni-NTA affinity chromatography followed by ion-exchange chromatography (2Q and 3SP columns), yielding a final product with a purity of ≥90% [73]. Wang et al. [20] employed nickel affinity column chromatography to purify the recombinant human collagen α1(III) chain, collecting the first 2 mL of eluate for analysis by SDS-PAGE, though no specific purity value was reported. Zhao et al. [69] found that adding a SUMO tag to the N-terminus of the protein significantly enhanced its stability and effectively improved its bioactivity.

Although affinity chromatography is an efficient and specific purification technique, it still has several limitations in practical applications, especially in the preparation of complex recombinant proteins. First, the His-tag fused at the N- or C-terminus may not be effectively exposed due to steric hindrance, reducing its binding efficiency to the nickel column. Second, customizing high-affinity ligands requires additional steps for protein expression and purification, as well as coupling to the solid phase, which increases the complexity and cost of the overall process. Finally, the fusion tag may affect both the protein’s structure and its biological activity [74].

### 3.3. Ion Exchange Chromatography

**Figure 6:**
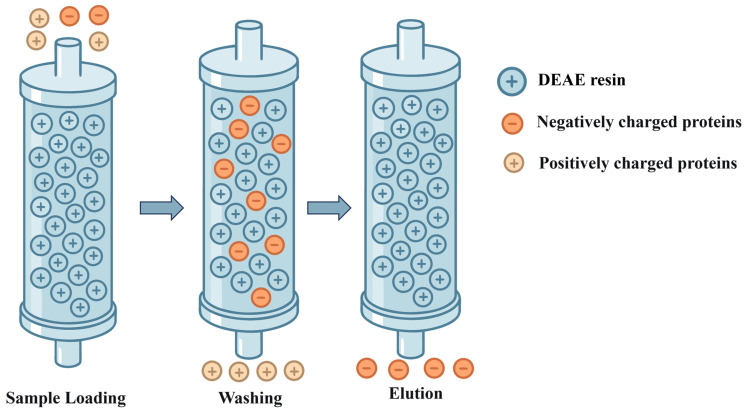
Schematic Diagram of Anion Exchange Chromatography.

The principle of ion exchange chromatography relies on the reversible adsorption and separation of target molecules through electrostatic interactions between the molecules and the charged functional groups on the chromatographic medium [75] (Figure 6).

Ion exchange chromatography is a common technique for protein purification [76,77]. The pH was adjusted to 4.0 with acetic acid, followed by SP FF ion-exchange chromatography, which achieved high-purity recombinant collagen with an excellent recovery yield [57]. In recent years, to meet purification requirements and achieve higher purity, the combined use of affinity chromatography followed by cation exchange chromatography has become increasingly common. Xu et al. [30] employed a two-step purification process involving nickel affinity chromatography and SP cation exchange chromatography, this strategy increased the purity of recombinant type III collagen from 89.60% (affinity chromatography peak P1) to 95.44% (SP2-2 elution fraction), meeting the requirements for high-purity medical-grade raw materials. Similarly, Teng et al. [72] reported that a two-step purification process involving nickel affinity chromatography and anion exchange chromatography increased the purity of recombinant type I collagen (α1 chain) from over 50% after the initial step to over 95% in the final product, satisfying the criteria for high-purity protein. The recombinant collagen was purified using cation-exchange chromatography, yielding high-purity protein suitable for structural and functional characterization [78].

Ion exchange chromatography is a key step in obtaining high-purity recombinant collagen due to its high resolution, ease of scale-up, and low cost. However, its limitations are notable. The method imposes stringent requirements on sample pretreatment; prior to loading, the sample conductivity often needs to be drastically reduced via dilution, ultrafiltration, or desalting, and the pH environment must be precisely controlled to ensure effective binding of the target protein. This process significantly increases operational complexity and time costs. Furthermore, its efficacy depends on the surface charge distribution of the target protein versus impurities, resulting in limited separation efficiency for molecules with similar charge characteristics.

### 3.4. Gel Filtration Chromatography

**Figure 7:**
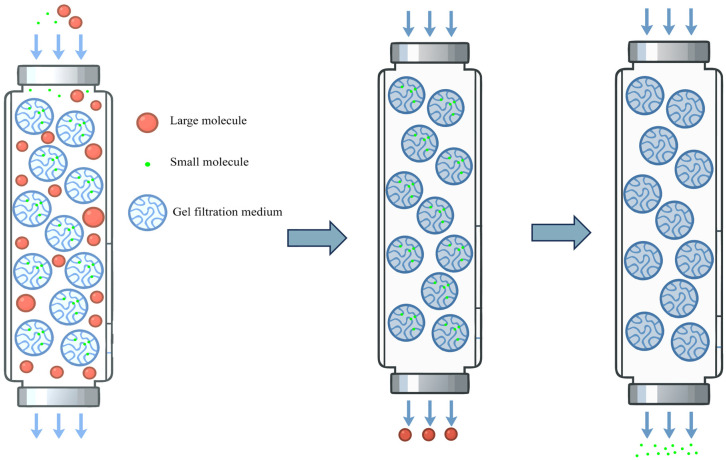
Schematic diagram of Gel Filtration Chromatography.

Gel filtration chromatography, also known as molecular sieve or size-exclusion chromatography, separates mixture components based on the size (hydrodynamic volume) of the molecules [79] (Figure 7).

The recombinant COL17A1 fragment was purified by cation-exchange chromatography followed by desalting and lyophilization, though the final purity percentage was not explicitly stated in the reported results [80]. Based on a theoretical molecular weight of 21 kDa for the transdermal peptide–hCOL3A monomer and a size-exclusion chromatography result of 66 kDa, it is confirmed that the fusion protein exists as a trimer in buffer A [81]. Gel filtration chromatography revealed that rRABV-G-XVIII was predominantly trimeric (~250 kDa, 96.3%), with a minor monomeric peak (~61 kDa) [82]. Analysis by gel filtration chromatography determined the purity to exceed 98% [57]. Additionally, gel filtration is effective for separating high concentrations of imidazole or salt from elution buffers used in prior affinity or ion exchange chromatography steps, thereby serving as a desalting method.

As a purification technique based on molecular size, gel filtration chromatography offers low resolution. While it can effectively separate molecules with significantly different sizes during recombinant collagen purification, its efficiency is limited for proteins of similar sizes. Consequently, it is often used in combination with other chromatographic methods in practical applications to improve purification efficiency and product recovery. Gel filtration also imposes strict requirements on sample load volume, typically limited to 1–5% of the column bed volume, resulting in low throughput which is not conducive to large-scale preparation.

## 4. Characterization of Recombinant Collagen

Systematic structural characterization and bioactivity validation of recombinant collagen are indispensable scientific foundations for its transition from molecular design to industrial application. Structural characterization forms the basis for elucidating its structure-activity relationship. Techniques such as circular dichroism spectroscopy and spectral analysis confirm the presence of its functional structure (Table 4), thereby ensuring its structural biomimicry of natural collagen at the molecular level, which serves as a prerequisite for specific cellular recognition and interactions. Bioactivity characterization, on the other hand, is crucial for evaluating its functional efficacy. It requires confirmation through in vitro and in vivo cellular experiments that it can guide the behavior of target cells and simulate the physiological functions of the extracellular matrix, thereby promoting tissue repair and regeneration.

### 4.1. Physicochemical Properties

**Figure 8:**
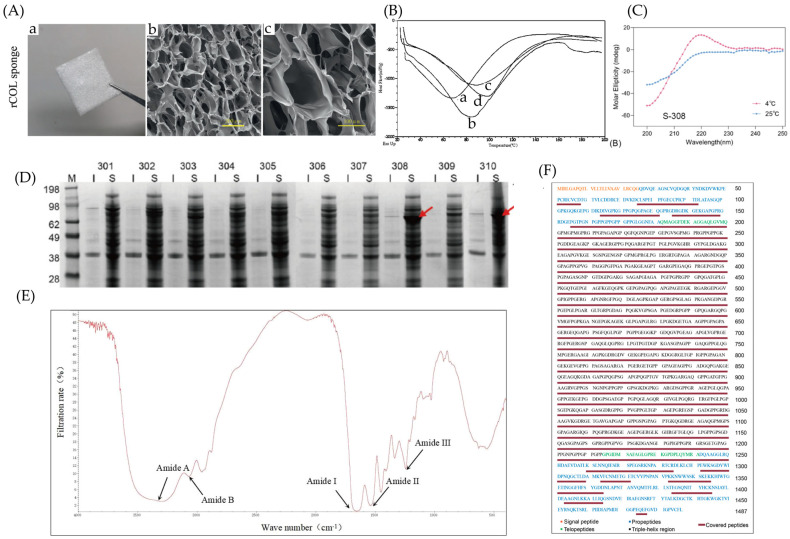
Schematic summary of physicochemical characterization methods for recombinant collagen. (**A**) The electron microscopy of natural collagen sponge also showed 3D network structure with tight pore structure. (**a**) Morphology of rCOL sponge; (**b**,**c**) microstructure of rCOL sponge [83]. (**B**) The measured DSC data showed that by increasing HA content in collagen-based materials enhanced the effectiveness of the cross-linking reaction and intermolecular linkages leading to a better overall stability. (a) rCOL (0% HA); (b) rCOL (2.5% HA); (c) rCOL (5% HA); (d) rCOL (10% HA) [84]. (**C**) A positive elliptical band in the range of 220–222 nm on the standard curve indicates the formation of a triple helix structure [85]. (**D**) The selected fragments were amplified 16-fold and successfully expressed in a soluble form by using a TrxA/His dual tag system. The red arrow indicates the target protein [85]. (**E**) FTIR spectrum of r-HLC-Zn complex had some differences in functional groups compared with free r-HLC, which indicated that both groups of C=O and N-H are involved in the metal-ligand formation, and the resultant complex remains the helical structure as r-HLC does [86]. (**F**) The sequence coverage confirmed by the MS data was 86.35%, with complete coverage (100%) of the triple-helix region [23].

As a revolutionary substitute for animal-derived collagen, the industrial application potential of recombinant collagen highly depends on the systematic analysis of its hierarchical structural stability and biofunctional reliability [40]. At the level of physicochemical properties: SDS-PAGE electrophoresis verifies the target protein’s molecular weight and purity; Scanning Electron Microscopy (SEM) reveals the three-dimensional surface morphology of lyophilized recombinant collagen; Micro Differential Scanning Calorimetry quantifies the thermal denaturation temperature to assess the stability of the triple-helical structure; Fourier Transform Infrared Spectroscopy (FTIR) confirms the primary structure via characteristic peaks; Circular Dichroism Spectroscopy characterizes the characteristic negative and positive peaks at the molecular level to confirm the presence of the triple-helical structure; Mass Spectrometry matches the amino acid sequence coverage between the theoretical and experimentally determined sequences (Figure 8).

#### 4.1.1. SDS-PAGE Electrophoresis

**Figure 9:**
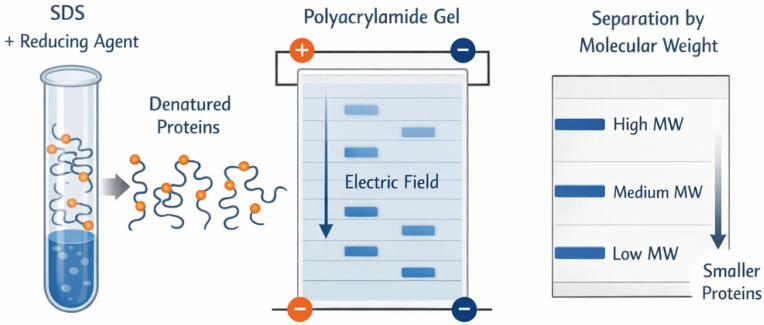
Schematic diagram of SDS-PAGE.

SDS-PAGE is a fundamental analytical technique in molecular biology and biochemical research. This method separates protein mixtures based on the different migration rates of protein molecules under an electric field according to their relative molecular masses (Figure 9). In recombinant collagen research, SDS-PAGE can determine the expression level of the target protein, its molecular weight distribution characteristics, and purity.

SDS-PAGE analysis clearly identified that among five rationally designed collagen variants (ColP1~ColP5), only ColP2 and ColP5 were successfully secreted into the fermentation supernatant, with apparent molecular weights of approximately 65 kDa and 80 kDa, respectively, matching theoretical predictions [29]. Multiple studies have observed [87] that recombinant collagen in the fermentation supernatant primarily exists as dimers, along with a smaller proportion of monomers. Through optimization of fermentation temperature, IPTG concentration, and incubation time, the highest expression level of rhColIV-E (2.06 g/L) was achieved at 25 °C with 0.6 mM IPTG after 20 h of induction [71]. A notable phenomenon during SDS-PAGE analysis of recombinant humanized collagen is that its apparent molecular weight is often larger than the theoretical. All screened clones expressed rhCOL3A1 with an apparent molecular weight of 130 kDa, which is higher than its theoretical molecular weight of 96 kDa [27]. This is speculated to be due to the high hydrophilicity of recombinant humanized collagen, which reduces its binding affinity to SDS, leading to slower electrophoretic migration and an overestimation of molecular weight [88].

In the characterization of recombinant collagen, SDS-PAGE electrophoresis offers the advantages of being rapid and intuitive for assessing protein purity, estimating molecular weight, and detecting degradation fragments, coupled with simple operation and low cost. However, its main disadvantages are that it must be performed under denaturing conditions, cannot reflect the native triple-helical structure or biological activity, and has limited resolution for impurities with similar molecular weights or highly glycosylated proteins. This technique is primarily used as a basic qualitative tool for rapid purity monitoring at various stages of the purification process and for preliminary molecular weight estimation. However, confirming the identity of the target protein requires combination with methods such as Western blot or LC-MS/MS for more precise quality control analysis.

#### 4.1.2. Electron Microscopy Analysis

**Figure 10:**
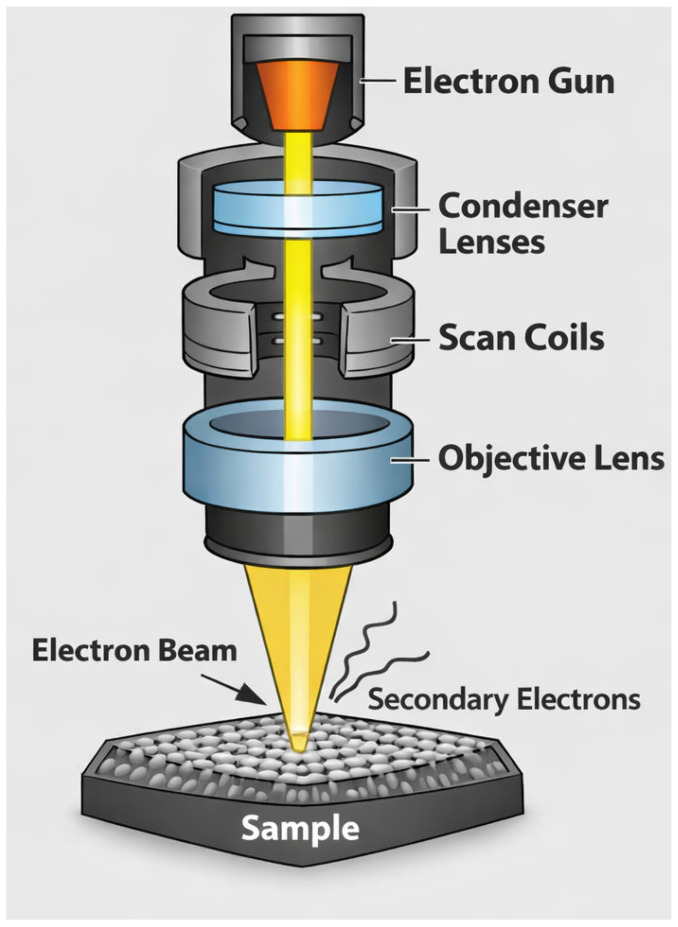
Schematic diagram of SEM.

Scanning Electron Microscopy (SEM) can observe the micro-morphology and porous structure, assess the fiber assembly state, or analyze the quality of the lyophilization process by scanning the surface morphology of recombinant collagen and its crosslinked products with an electron beam [89] (Figure 10). Besides SEM, which is the most frequently used, Transmission Electron Microscopy (TEM) can also examine the internal structure of nanofibers formed by the self-assembly of recombinant collagen, particularly their diameter, arrangement, and the characteristic periodic cross-banding, to verify if it correctly mimics the core structure of natural collagen.

Different porous structures of biomaterials can lead to different biological effects [90].

Currently observed lyophilized samples of recombinant collagen often exhibit filamentous and pore-like structures, fibrous structures, or network structures. Pore sizes above 100 µm facilitate vascular ingrowth into the tissue, and pore sizes above 200 µm exhibit superior osteogenic efficacy, meeting the requirements for pore size in bone repair materials. Tong et al. [91] observed via SEM that recombinant double-humanized collagen had a filamentous morphology with numerous pore-like structures, and the structure size was relatively uniform. Dong [92] observed that after freeze-drying, recombinant human-like collagen and recombinant human-like fibronectin formed distinct but irregular network structures. Different micro-morphologies suggest potential application scenarios. 

Wang et al. [20] observed that the fibrous structure formed through the aggregation of thinner fibrils and exhibited the characteristic D-band periodicity. TEM analysis confirmed that exosomes isolated from human bone marrow-, adipose-, and umbilical cord-derived mesenchymal stem cells exhibited a characteristic bilayer membrane structure with a discoid shape, intact morphology, and uniform size distribution [93]. Yu et al. [94] observed that hydroxyapatite crystals were uniformly dispersed in the recombinant type II collagen/chitosan matrix, with some nanoparticles adsorbing together.

During sample preparation for SEM and TEM, recombinant collagen composite materials may lead to the collapse of hydrogel network structures, agglomeration of nanoparticles, or changes in the crystal morphology of composites, failing to accurately reflect the material’s microstructure in its original hydrated state. This can be complemented by methods such as Atomic Force Microscopy (AFM) and Cryo-Electron Microscopy to observe the structure in a state closer to the native one, supplemented by quantitative image analysis to enhance the scientificity and reliability of the data.

#### 4.1.3. Thermal Analysis

**Figure 11:**
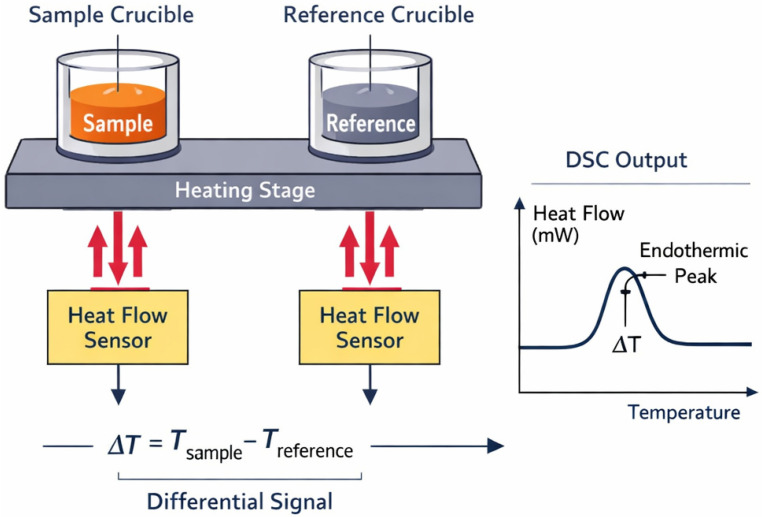
Schematic diagram of DSC.

DSC (Differential Scanning Calorimetry) is a thermal analysis technique that detects thermal transitions in materials by measuring the heat flow difference between a sample and a reference under programmed temperature control (Figure 11). The thermal stability of recombinant collagen and its composite materials can be assessed using Differential Scanning Calorimetry (DSC) and Thermogravimetric Analysis (TGA).

Cheng et al. [95] found via DSC analysis that the denaturation temperature of recombinant type III collagen significantly increased after binding with epidermal growth factor. As the content of cross-linking material increased, the thermal denaturation temperature of the material rose significantly, suggesting that increasing the cross-linker content can enhance the overall thermal stability of the composite material [84]. Differential scanning calorimetry (DSC) analysis revealed that the thermal denaturation temperature (T_max_) of RhCol2 was 40.5 °C [23]. The structural variations generated by the crosslinking process were also evaluated by TGA, cross-linking treatment significantly enhanced the thermal stability of collagen materials, resulting in a reduced degradation rate and increased residual mass at elevated temperatures, while the functionalization with ICOS-Fc did not notably alter this thermal stability profile [96].

While DSC or TGA can provide macroscopic information on the overall thermal behavior of a sample, they cannot reveal the specific molecular mechanisms or structural details causing denaturation or degradation. DSC measurements are susceptible to interference from factors such as moisture in the sample, cold crystallization, or molecular rearrangement, leading to overlapping of the thermal denaturation peak with irreversible endothermic events like water evolution, shrinkage, or depolymerization, complicating the accurate determination of collagen’s thermal denaturation temperature and potentially leading to misjudgment of thermal stability. TGA primarily reflects weight changes, but the weight loss in recombinant collagen often stems from the release of bound or non-crystalline water rather than protein decomposition itself. This can mask the true thermal stability and fails to provide information on structural changes.

#### 4.1.4. Circular Dichroism Spectroscopy

**Figure 12:**
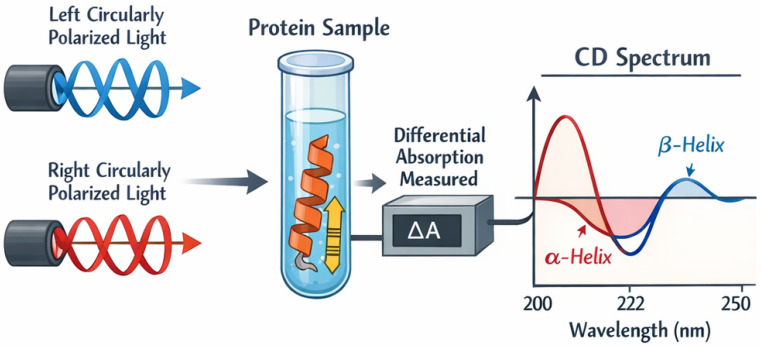
Schematic diagram of CD.

Circular Dichroism (CD) spectroscopy, based on the principle of differential absorption of left and right circularly polarized light [97] (Figure 12), can be used to evaluate the secondary structure, binding properties, and folding state of recombinant collagen.

RhCol1 shows a CD spectrum very close to that of human type I collagen, with a positive band at 221 nm and a negative band at 197 nm, indicating a triple helical structure [20]. COL3 fragments exhibited positive absorption peaks at 220–222 nm, but as the temperature increased to 25 °C, these peaks disappeared, indicating a loss of triple helix formation ability at this temperature [85]. In their CD spectroscopic analysis, Chang et al. [70] observed that rhColIV-E exhibited a pronounced negative peak at 200 nm but showed no discernible positive peak at 220 nm, indicating the absence of a triple-helix structure, and further revealed that its secondary structures consist predominantly of β-antiparallel sheets and random coils. Liu [98] found that the secondary structure of recombinant human collagen consisted of 21.3% α-helix, 32.5% β-sheet, 23.2% turns, and 23.0% random coil, indicating a structure similar to natural collagen. These studies indicate that a positive peak around 220 nm in CD spectroscopy is a key diagnostic feature for identifying the triple-helix structure of recombinant collagen.

CD analysis primarily provides overall structural information about the overall structure and cannot resolve local conformations. Without combination with other structural validation methods to further confirm its higher-order structure, it has certain limitations in determining whether recombinant collagen possesses the native structure and function.

#### 4.1.5. Mass Spectrometry Analysis

**Figure 13:**
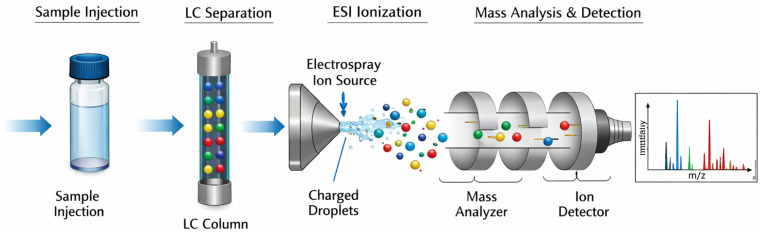
Schematic diagram of MS.

Mass spectrometry works by converting recombinant collagen molecules into charged ions and separating and detecting them based on their mass-to-charge ratio (*m*/*z*), allowing for the analysis of amino acid sequence coverage and molecular weight [99] (Figure 13).

Cui et al. [100] determined the relative molecular mass of recombinant humanized type III collagen using Time-of-Flight Mass Spectrometry and Size Exclusion Chromatography–Multi-Angle Light Scattering, and performed primary structure analysis via a double-enzymatic peptide mapping method, which confirmed consistent molecular mass values, 100% amino acid sequence coverage, and agreement between the experimental and expected sequences. Xiang [101] used mass spectrometry to analyze the purified recombinant collagen and found that the experimental molecular weight was completely consistent with the theoretical value. Through the co-expression of R8 with Bacillus anthracis prolyl 4-hydroxylase (BaP4H) in EcN, a hydroxylation level of 60 % was achieved, and to precisely map the hydroxylation sites, the purified products were analyzed by LC-MS/MS [102].

In the characterization of recombinant collagen, mass spectrometry enables highly specific, sensitive, and accurate qualitative and quantitative analysis, precisely determining amino acid sequence coverage, post-translational modifications (such as proline hydroxylation, glycosylation), and degradation fragments. However, this method requires sophisticated instrumentation, involves complex operation and high costs, and is susceptible to interference from complex components when analyzing low-abundance recombinant collagen in complex formulations.

#### 4.1.6. Fourier Transform Infrared Spectroscopy

**Figure 14:**
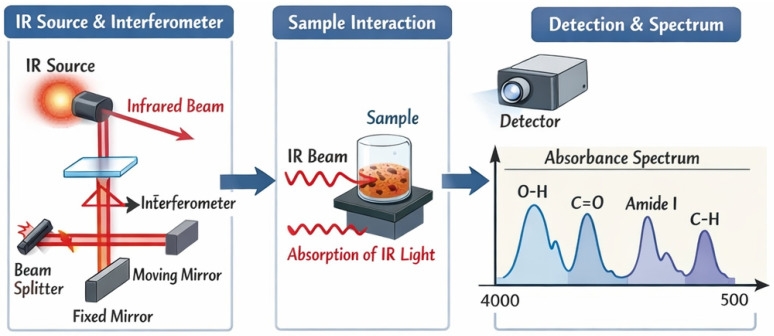
Schematic diagram of FTIR.

The fundamental principle of FTIR is based on atomic vibrations and rotations [103] (Figure 14). It can assess the structural similarity between recombinant collagen and natural collagen by detecting characteristic absorption bands, primarily Amide A (3300–3500 cm^−1^), Amide B (~3100 cm^−1^), Amide I (1600–1800 cm^−1^), Amide II (1470–1570 cm^−1^), and Amide III (1250–1350 cm^−1^) [104,105].

Beyond assessing structural similarity to natural collagen, FTIR can also be used to judge the integrity of recombinant collagen composite materials. The FTIR results confirmed the successful modification of collagen, as evidenced by a distinct change at 950 cm^−1^ (corresponding to the out-of-plane bending vibration of RC=CH_2_ in rhCol-MA), along with a blue-shifted C=C stretching vibration at 1698 cm^−1^ in CMC-GMA, further verifying the incorporation of GMA [106]. FTIR analysis confirmed the successful grafting of TK-NH_2_ onto rhCOL17, demonstrating the formation of rhCOL17-PTK [107]. He et al. [84] found that the addition of crosslinkers did not significantly alter the spectral features, indicating no introduction of other chemical groups during the cross-linking reaction and the maintenance of the original chemical structure, confirming the chemical stability of the cross-linking process. Yu et al. [108] found differences in functional groups between the recombinant human-like collagen-zinc complex and free recombinant human-like collagen, suggesting that both C=O and N-H groups participated in metal–ligand formation, while the resulting complex maintained the same helical structure as recombinant human-like collagen. These studies demonstrate that FTIR analysis can effectively confirm the successful modification and grafting of collagen, verify the chemical stability of cross-linking, and characterize the formation of metal-ligand complexes while preserving the original chemical

Although FTIR plays an important role in functional group identification, its sensitivity and quantitative capability in reactions with low degrees of substitution are limited, necessitating combination with other analytical techniques for comprehensive characterization of the material structure.

### 4.2. Biological Function

**Figure 15:**
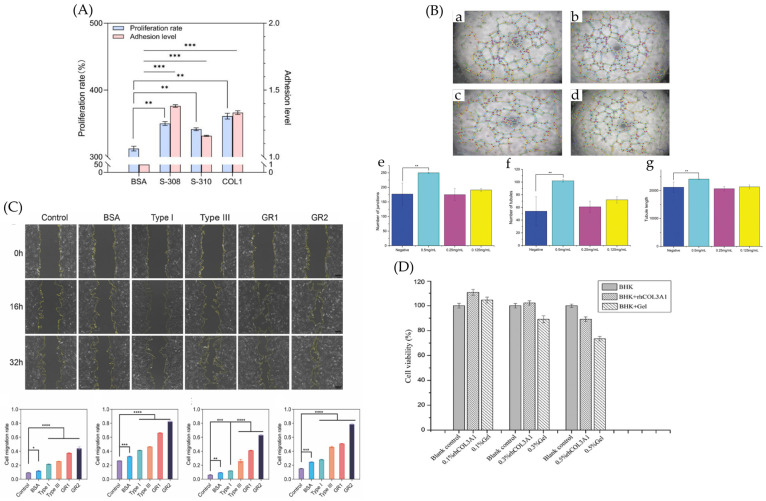
Overview of the in vitro bioactivity of recombinant collagen. (**A**) The cell adhesion rate of COL1, sample-308, and sample-310 was higher than that of BSA, with sample-308 exhibiting the best cell adhesion effect, which was 30% higher than that of BSA [85]. (**B**) HCOLIII can promote blood vessel formation at higher concentrations. (**a**–**d**) Promoting effect of different concentrations of hCOLIII on angiogenesis; (**e**–**g**) three key indicators in vascular formation experiments: Number of junctions, Number of tubules, Tubule length [113]. (**C**) Compared with the control, the addition of GR1 and GR2 proteins in the experimental group had a more pronounced effect on HSF migration, especially the addition of GR2 protein, which significantly enhanced the migration of HSF. The reduction in the gap between the two yellow lines represents the distance of cell migration over time [109]. (**D**) Low concentration of rhCOL3A1 solution could effectively promote BHK21 cell proliferation, and the activity of rhCOL3A1 promoting cell proliferation was obviously higher than gelatin from porcine skin [27]. * *p* < 0.05, ** *p* < 0.01, *** *p* < 0.001, **** *p* < 0.0001.

**Figure 16:**
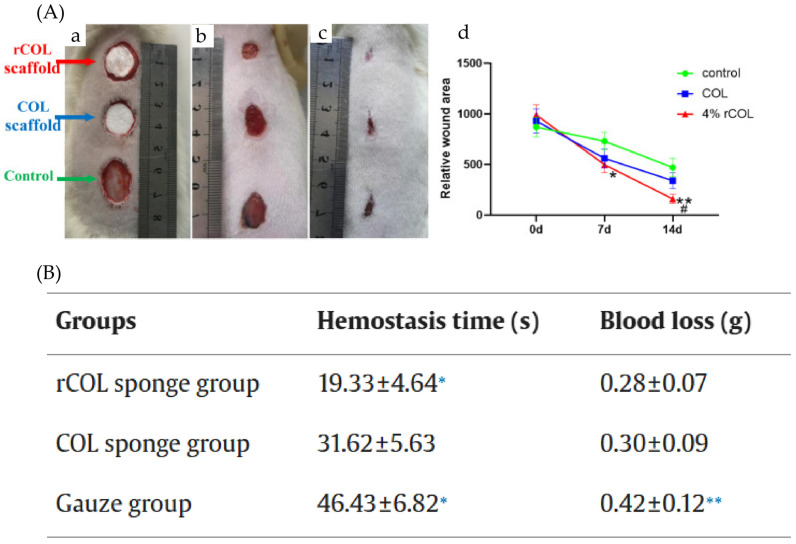
Overview of the in vivo bioactivity of recombinant collagen. (**A**) With crosslinked rCOL scaffolds showing significantly greater efficacy compared to animal-derived collagen 14 days post-treatment. (**a**) Immediately, (**b**) 7 days, (**c**) 14 days, the wound repair status of full-layer skin defect on the back of rats; (**d**) skin defect area histogram at different time points [110]. (**B**) The rCOL sponge group exhibited a shorter hemostasis time compared to the control group [116]. (* *p* < 0.05, ** *p* < 0.01, compared with control group; # *p* < 0.05, compared with COL group).

Through rational design and optimization of the functional sequences of recombinant collagen, along with the incorporation of specific functional domains, it demonstrates significantly enhanced biological activity—including in cell proliferation, migration, adhesion, vascularization, wound healing, and hemostasis—surpassing that of conventionally extracted animal-derived collagen (Table 5). In vitro biological function analysis aims to assess its biocompatibility and regulatory effects on specific cellular behaviors using in vitro cell model systems. This includes key biological effects such as cell adhesion, spreading, migration, proliferation, and the induction of extracellular matrix synthesis, thereby validating its bioactivity and application potential (Figure 15). In vivo functional analysis evaluates its post-implantation biocompatibility, ability to repair and regenerate wounded or defective tissues, and overall safety in live animal models, to verify its actual efficacy in complex physiological environments (Figure 16).

#### 4.2.1. In Vitro Functional Analysis

Cell receptors related to collagen include integrins, receptor tyrosine kinases, immunoglobulin receptors, leukocyte receptor complexes, fibronectin, and vitronectin [111]. As an important component of the extracellular matrix, collagen provides adhesion sites for cells, primarily because cells bind to collagen via surface integrins, thereby achieving adhesion [112]. Integrins, being key receptors mediating cell adhesion and signal transduction, have become a research hotspot in the field of recombinant collagen.

Recombinant α1(IV)NC1 and α2(IV)NC1 domains restored proliferation in MT2-siRNA-treated SMG cells, and the C-terminal non-collagenous (NC1) domain could promote branching morphogenesis by regulating integrin-mediated cell adhesion, modulating cell migration, and influencing extracellular matrix degradation [113]. The adhesion of human neural stem/progenitor cells (hNSPCs) to recombinant collagen containing the GFOGER sequence is primarily mediated by the α1β1 integrin heterodimer, as evidenced by the complete inhibition of adhesion with anti-β1 antibodies and its significant reduction with anti-α1 antibodies [114]. In another experiment, inhibition studies using monoclonal antibodies against integrin α1 and α2 subunits further confirmed the role of integrins α1β1 and α2β1 in cell adhesion to collagen XVI [115]. He [116] found that recombinant collagen, after better amino acid sequence optimization, possessed stronger biological activity compared to natural collagen sponges. However, the bioactivity of recombinant collagen is often inferior to that of natural collagen. In cell proliferation experiments, cell viability was only about 20% of that with natural type I collagen [117]. Natural collagen-modified hydrogels showed the highest cell adhesion rate at 51%, synthetic collagen peptide GFOGER-modified hydrogels showed 47%, while recombinant collagen-modified hydrogels showed a cell adhesion rate of 36% [118]. The adhesion capability of human neural stem cells to recombinant collagen substrates was related to the density of GFOGER sequences on the substrate.

In the characterization of recombinant collagen, cell proliferation experiments serve the core purpose of functionally validating its bioactivity. They directly reflect the ability of recombinant collagen to support cell growth and maintain basic physiological functions. The effect of recombinant collagen on cell proliferation can typically be measured using the CCK-8 [119] or MTT [120] assay. Recombinant humanized collagen significantly promoted epidermal cell growth activity and cell migration, showing a certain concentration dependency [83]. The CCK-8 assay demonstrated that rhCol III hydrogel promotes the proliferation of HUVECs [121]. Zhou et al. reported that treatment with different concentrations of the RHC complex for 16 h significantly enhanced cell viability, and this effect was concentration-dependent [122]. The MTT assay in Study 2 showed excellent cytocompatibility, with L929 fibroblast viability exceeding 100% after 24 h exposure to the hydrogel extract in a diabetic wound-like environment [123]. Collectively, these studies demonstrate that recombinant humanized collagen significantly enhances the proliferation, viability, and migration of various cell types in a concentration-dependent manner.

The scratch assay simulates cell migration during wound healing by creating a scratch and observing the migration speed of cells in recombinant collagen-treated groups versus control groups [124]. Multiple in vitro experiments have shown that recombinant collagen can significantly promote cell migration and scratch closure. The multi-network hydrogel, especially the GHrCT1 formulation, effectively maintained the migration ability of L929 mouse fibroblast cells under oxidative stress, due to its ROS-scavenging property [119]. Recombinant type III collagen thermo-sensitive hydrogel significantly promoted the migration of human endometrial stromal cells (hESCs), demonstrating its potential for endometrial repair [120]. The wound gap significantly decreased after 48 h of treatment with two recombinant collagen fragments, confirming the important role of recombinant type I collagen in promoting the migration and adhesion of skin-derived cells [11].

To verify the pro-angiogenic activity of recombinant collagen, a classic in vitro tube formation assay was conducted. This assay involved seeding human umbilical vein endothelial cells on Matrigel and quantitatively analyzing key morphological indicators, such as the total capillary length, number of meshes, junctions, and segments, to objectively reflect the formation of vascular networks. Recombinant type III collagen effectively promoted the tube-forming capability of human umbilical vein endothelial cells, as evidenced by significant increases in total capillary length, number of meshes, junctions, and segments [120]. In vitro, the extract of the dual-network hydrogel significantly promoted the tube-forming capacity of human umbilical vein endothelial cells (HUVECs), as evidenced by increased number of nodes, branches, and average tube length compared to controls [106].

#### 4.2.2. In Vivo Functional Analysis

Recombinant collagen has been extensively studied in wound healing across various scenarios.

In a rat deep second-degree burn model, recombinant collagen-chitosan hydrogel injected into the burn site resulted in a significantly higher wound closure rate compared to the control group, promoting cell infiltration, angiogenesis, and wound healing, indicating its potential to promote wound healing [125]. In a rat model of chemotherapy-induced oral mucositis, wounds treated with recombinant collagen composite material healed significantly faster with a notably reduced wound area, and exhibited enhanced angiogenesis and collagen repair in the early stages [126]. In a full-thickness skin defect model, recombinant collagen hydrogel significantly shortened the healing cycle (by over 30%) and accelerated wound healing speed [127] and histological staining results showed more mature skin tissue repair, the appearance of new hair follicles, and higher collagen fiber content in the recombinant collagen hydrogel group. In a rat calvarial defect model, recombinant collagen hydrogel significantly promoted new bone formation, accelerated bone regeneration, markedly reduced the defect size, and resulted in abundant formation of new bone tissue [128]. In a diabetic mouse wound model [129], the experimental group containing recombinant collagen protein showed a significantly higher wound healing rate than other groups, along with significantly increased expression of COL-1α and Cytokeratin-14 (CK-14) at the wound site, indicating its significant effect in promoting diabetic wound healing. Across different experimental models, recombinant collagen consistently demonstrated significant repair effects. These results collectively indicate that its function is no longer limited to traditional physical filling and barrier support but extends to active regulation, enabling recombinant collagen to orderly guide host cell behavior and collectively shape a microenvironment conducive to tissue healing.

The purpose of hemostasis experiments for recombinant collagen composites is to assess their efficacy in controlling acute hemorrhage in vivo. This is typically measured by key parameters such as total blood loss and time to hemostasis in standardized injury models. In a mouse tail-transection model, the recombinant collagen fragment/hyaluronate composite demonstrated superior hemostatic efficacy, reducing bleeding by >50% compared to commercial collagen I [85]. In a rat liver hemorrhage model, the dual-network hydrogel significantly reduced blood loss and shortened hemostasis time compared to untreated controls, matching or surpassing a commercial hemostatic agent [106]. Collectively, these findings confirm that recombinant collagen-based composites can achieve rapid and effective hemostasis in vivo.

## 5. Summary and Outlook

As an important biomaterial, recombinant collagen offers numerous advantages over traditional collagen. Its outstanding bioactivity and antioxidant capacity form the basis of its biological efficacy. Meanwhile, its inherent freedom from viral risks and low immunogenicity provide favorable compatibility for clinical translation. Furthermore, its good water solubility ensures compatibility and efficient delivery within biological systems, while mature and stable production processes offer a solid foundation for large-scale, high-quality manufacturing. Building upon these inherent strengths, recombinant collagen holds promising prospects for broader applications in fields such as tissue engineering, regenerative medicine, and drug delivery in the future.

Recombinant expression systems such as *E. coli* are widely used as mainstream platforms due to their high efficiency and low cost, but they still face challenges in achieving proper post-translational modifications and folding of collagen proteins. Eukaryotic expression systems such as *P. pastoris* and mammalian cells can perform post-translational modifications but suffer from drawbacks like high cost and long cycles. Insect and plant expression systems can express relatively complex proteins, but their expression levels are relatively low. The *E. coli* and *P. pastoris* expression systems are currently relatively well-established for industrial-scale production. However, a key limitation in using these systems for recombinant collagen production is their inherent lack of endogenous hydroxylation capacity—specifically, the absence of prolyl 4-hydroxylase (P4H) activity. This modification is naturally provided by mammalian expression platforms. For *P. pastoris*, this shortfall can be addressed through engineering strategies such as co-expression of P4H enzymes, highlighting the distinction between its native state and its engineered potential. Moving forward, reducing mammalian cell culture costs and improving their expression yields could help overcome current constraints, thus promoting wider industrial application of mammalian systems for recombinant collagen manufacturing.

Regarding separation and purification techniques, methods such as precipitation, affinity chromatography, ion-exchange chromatography, and gel filtration chromatography each possess distinct characteristics. Their selection and combination should be rationally tailored to specific requirements to achieve efficient purification. Due to significant differences in host cell protein profiles arising from different recombinant collagen expression systems, purification processes must be developed and optimized on a case-by-case basis; no universal purification strategy exists. Furthermore, production-scale chromatography resins—particularly those from imported brands—are costly and constitute a major portion of overall production expenses. Therefore, a key ongoing challenge for manufacturers is to continuously optimize processes to ensure high purity while improving binding capacity, yield, and cost control.

Advances in characterization techniques have provided strong support for the identification and functional evaluation of recombinant collagen. Physicochemical analyses reveal its structural characteristics and thermal stability, while biological functional assays further validate its biocompatibility and potential for applications such as tissue repair. However, techniques such as FTIR and CD primarily reflect the overall proportions of secondary structures. They cannot directly determine finer structural features, such as correct folding, periodicity, and hydroxylation degree of the triple-helical structure, which require comprehensive characterization through integration with other analytical methods. Moreover, biological activity studies of recombinant collagen often lack mechanistic insights, and a clear quantitative correlation between in vitro activity data and in vivo repair efficacy or clinical outcomes has yet to be fully established. In the future, combining molecular biology techniques to explore the specific signaling pathways involved in its reparative functions will be essential for gradually advancing toward clinical research.

Overall, although significant progress has been made in recombinant collagen research and industrialization is gradually maturing, several challenges remain in its current development. Based on this, some future perspectives for recombinant collagen development are proposed.

(1) Research and application of recombinant collagen still face technical challenges. There is a lack of highly efficient expression systems capable of producing recombinant collagen with advanced modifications, and the expressed single chains often struggle to self-assemble into higher-order structures. Future studies should focus on developing more efficient expression systems for recombinant collagen and delving deeper into its self-assembly mechanisms, aiming to achieve efficient production of highly modified collagen and enable controlled construction of its advanced structures.
(2) The commercial application of recombinant collagen is primarily limited by its production costs. Low efficiency in gene sequence and protein structure design, requiring repeated trial and error, increases R&D costs. In the future, AI large models (e.g., AlphaFold) can assist in design, accurately predicting three-dimensional structures to enable function-oriented optimization, effectively improving the accuracy and success rate of recombinant protein design, thereby reducing R&D costs. Complex purification steps and expensive purification media also contribute to high R&D costs. Future efforts should focus on developing simpler purification processes and more cost-effective purification media to reduce purification costs.
(3) In recent years, the application of recombinant collagen in the cosmetics field has developed rapidly, but it lacks standardized testing methods and authoritative evaluation systems, leading to product quality controversies. It is imperative to establish and improve standard testing methods and authoritative evaluation systems for recombinant collagen.
(4) Clinical translation faces issues such as a lack of long-term safety data, limited indications, and incomplete assessment of immunogenic risk. Future development should focus on building a systematic long-term safety evaluation system and deepening the exploration of immunological mechanisms and precise indications.
(5) Current research on recombinant collagen mainly focuses on types I, II, III, and XVII. Moving forward, priority should be given to the development of recombinant human type IV, V, and VII collagen. Type IV collagen serves as a key component of the skin’s basement membrane, with well-established applications in high-end repair and anti-aging treatments. Type V collagen is indispensable for constructing highly biomimetic and transparent artificial corneal stroma. Type VII collagen can be used directly to treat life-threatening rare diseases such as dystrophic epidermolysis bullosa, for which there is an urgent clinical demand.

## Figures and Tables

**Table 1:** Hosts and Expression Levels for Different Types of Collagen.

Type of Collagen	Host	Expression Level	Year	Reference
Recombinant Type I Collagen	*E. coli*	500 mg/L	2016	[10]
		1.88 g/L	2023	[11]
		0.8 g/L	2023	[12]
	Yeast	0.5 g/L	2001	[13]
		4.5 g/L	2019	[14]
	Plant (Tobacco)	30 mg/kg	2000	[15]
		50~100 mg/kg	2002	[16]
		200 mg/kg	2009	[17]
	Plant (Corn)	Hydroxylated: 4 mg/kg; Unhydroxylated: 12 mg/kg	2011	[18]
	Mammalian cells (mammary cells)	8 g/L	1999	[19]
	Mammalian cells (CHO)	300 m g/L	2024	[20]
Recombinant Type II Collagen	Yeast	3.04 g/L	2024	[21]
	Insects (Sf9)	50 mg/L	1998	[22]
	Insects (Sf9)	1 mg/larva	2016	[23]
	Mammalian cells (CHO)	188.73 mg/L	2025	[24]
Recombinant Type III Collagen	*E. coli*	120 mg/L	2024	[25]
	Yeast	10.3 g/L	2025	[26]
		4.68 g/L	2015	[27]
		2.15 g/kg	1997	[28]
		2.54 g/L	2025	[29]
		3.5 g/L	2024	[30]
	Insects (Sf9)	40 mg/L	1996	[31]
	Insects (silkworm)	70 mg/silkworm cocoon	2003	[32]
Recombinant Type V Collagen	Yeast	4.36 g/L	2024	[33]
	Mammalian cells (293-EBNA)	15 mg/L	1997	[34]
Recombinant Type X Collagen	Mammalian cells (HEK293)	50 mg/L	1998	[35]
Recombinant Type XII Collagen	Yeast	4.89 g/L	2025	[36]
Recombinant Type I, III Collagen	Yeast	2.33 g/L	2022	[37]
	*E. coli*	1.36 g/L	2025	[38]
Hydroxylated Recombinant Collagen	*E. coli*	1.186 g/L	2024	[39]

**Table 2:** Comparative Analysis of the Characteristics of Recombinant Expression Systems.

Expression System	Advantages	Disadvantages
*E. coli*	Simple genetic manipulation; low cultivation cost; rapid growth rate; high expression levels; genetic stability; high transformation and transduction efficiency; suitable for large-scale production; highly commercialized.	Limited post-translational modifications; presence of endotoxins; potentially low protein activity.
*P. pastoris*	High safety profile; capable of certain post-translational modifications; correct protein folding; straightforward operation; well-established commercialization; high protein activity; good expression levels; low cultivation cost; scalable production.	Low secretion efficiency; presence of abundant proteases; requires methanol induction for expression; expression levels can be unstable.
Baculovirus–Insect Cell	Capable of complex post-translational modifications; correct protein folding; high protein activity.	High cost; cells are prone to lysis; susceptible to bacterial contamination; long growth cycle; generally low expression yields.
Mammalian Cell	High physiological activity; products closely resemble native proteins; capable of authentic post-translational modifications; stable cell lines available; ensures correct protein folding.	Very high cost; complex culture conditions; long growth cycle; susceptible to contamination; instability of transgene expression; labor-intensive operation; typically low expression yields.
Plant-Based System	High safety profile; capable of certain eukaryotic modifications; correct protein folding; relatively simple operation; high protein activity; good expression potential; scalable production; straightforward downstream processing.	Potential environmental biosafety concerns; long growth cycle of host plants; instability of transgene expression across generations.

**Table 3:** Comparison of Different Recombinant Collagen Purification Methods.

Purification Methods	Advantages	Disadvantages	Primary Application Scope	Reference
Precipitation	Very low cost, Simple to perform	Low specificity, risk of protein denaturation	Rough purification stage: For initial concentration of samples and removal of bulk impurities.	[59,60,61]
Affinity Chromatography	High selectivity and specificity, High recovery rate	High cost, Nonspecific binding	Fine purification: Ideal for proteins with specific tags	[62,63]
Ion Exchange Chromatography	High resolution and high capacity, Scalable	pI-dependent, Nonspecific binding	Rough to moderate purification: Suitable for separating most soluble proteins based on charge differences.	[64]
Gel Filtration Chromatography	Mild purification conditions, High recovery rate, Desalting capability	Limited resolution, Low capacity	Primarily used for desalting, buffer exchange, molecular weight determination	[65,66]

**Table 4:** Summary of Physicochemical Characterization Techniques for Recombinant Collagen.

Technique	Principle	Advantages	Disadvantages	Purpose in This Study
SDS-PAGE	Separates proteins based on molecular weight under an electric field	Simple, low-cost; assesses purity, MW, and subunits	Cannot provide precise MW or identify PTMs; requires denaturation	Check purity, polymerization state, and apparent MW
DSC	Measures the heat flow difference between a sample and a reference under programmed temperature control to detect thermal transitions	Directly determines thermal stability (Tm), requires small sample	Reflects global stability only; no specific structural insights	Evaluate triple-helix thermal stability vs. natural collagen
LM-MS	Ionizes sample molecules and separates/detects them based on their mass-to-charge ratio	Accurate MW, identifies PTMs, sequences	Expensive, requires high purity and expertise	Confirm MW and detect key proline/lysine hydroxylation
FTIR	Measures sample absorption of infrared light, reflecting the vibrational modes of chemical bonds	Fast, non-destructive; provides secondary structure info	Limited resolution in mixtures, overlapping bands, needs deconvolution	Confirm collagen-typical secondary structure
CD	Measures the difference in absorption of left- and right-handed circularly polarized light by proteins	Gold standard for solution secondary structure	Requires clear solutions, signal interference from aggregates/complexes	Quantify triple-helix content and monitor thermal unfolding
SEM	Scans the sample surface with a focused electron beam to detect secondary electrons for imaging	High-resolution surface morphology, porosity, and fiber arrangement	Destructive preparation (drying/coating); no chemical/internal information	Observe fibril network morphology or scaffold microstructure

**Table 5:** Biofunctional Performance of Recombinant Collagen In Vitro and In Vivo.

Biological Function	In Vitro/Vivo	Superior to Traditional Collagen	Source
Cell adhesion	In vitro	Cell adhesion rate: 50% higher than traditional collagen	[85]
Cell proliferation	In vitro	Cell viability: obviously higher than traditional collagen	[27]
Cell migration	In vitro	Mobility rate: generally higher than traditional collagen	[109]
Angiogenesis	In vitro	Vascular network density: higher than traditional collagen	[109]
Wound Healing	In vivo	Skin defect area: significantly greater efficacy compared to animal-derived collagen	[110]
Hemostasis	In vivo	Hemostasis time: > takes less time than traditional collagen.	[107]

## Data Availability

No new data were created or analyzed in this study.
